# Blood Amyloid Beta Levels in Healthy, Mild Cognitive Impairment and Alzheimer’s Disease Individuals: Replication of Diastolic Blood Pressure Correlations and Analysis of Critical Covariates

**DOI:** 10.1371/journal.pone.0081334

**Published:** 2013-11-27

**Authors:** Agustín Ruiz, Pedro Pesini, Ana Espinosa, Virginia Pérez-Grijalba, Sergi Valero, Oscar Sotolongo-Grau, Montserrat Alegret, Inmaculada Monleón, Asunción Lafuente, Mar Buendía, Marta Ibarria, Susana Ruiz, Isabel Hernández, Itziar San José, Lluís Tárraga, Mercè Boada, Manuel Sarasa

**Affiliations:** 1 Alzheimer Research Center and Memory Clinic, Fundació ACE, Institut Català de Neurociències Aplicades, Barcelona, Spain; 2 Araclon Biotech Ltd, Zaragoza, Spain; 3 Department of Psychiatry, Centro de Investigación Biomédica en Red en el Área de Salud Mental, Hospital Universitari Vall d'Hebron, Universitat Autònoma de Barcelona, Barcelona, Spain; 4 Hospital Universitari Vall d'Hebron, Institut de Recerca, Universitat Autònoma de Barcelona, Barcelona, Spain; CAEBi, Spain

## Abstract

Plasma amyloid beta (Aβ) levels are being investigated as potential biomarkers for Alzheimer’s disease. In AB128 cross-sectional study, a number of medical relevant correlates of blood Aβ40 or Aβ42 were analyzed in 140 subjects (51 Alzheimer’s disease patients, 53 healthy controls and 36 individuals diagnosed with mild cognitive impairment). We determined the association between multiple variables with Aβ40 and Aβ42 levels measured in three different blood compartments called i) Aβ directly accessible (DA) in the plasma, ii) Aβ recovered from the plasma matrix (RP) after diluting the plasma sample in a formulated buffer, and iii) associated with the remaining cellular pellet (CP). We confirmed that diastolic blood pressure (DBP) is consistently correlated with blood DA Aβ40 levels (r=-0.19, P=0.032). These results were consistent in the three phenotypic groups studied. Importantly, the observation resisted covariation with age, gender or creatinine levels. Observed effect size and direction of Aβ40 levels/DBP correlation are in accordance with previous reports. Of note, DA Aβ40 and the RP Aβ40 were also strongly associated with creatinine levels (r=0.599, P<<0.001) and to a lesser extent to urea, age, hematocrit, uric acid and homocysteine (p<0.001). DBP and the rest of statistical significant correlates identified should be considered as potential confounder factors in studies investigating blood Aβ levels as potential AD biomarker. Remarkably, the factors affecting Aβ levels in plasma (DA, RP) and blood cell compartments (CP) seem completely different.

## Introduction

Alzheimer’s disease (AD) is a global health problem for western countries, representing more than 60% of dementia cases in the world. The pathological findings of AD include the progressive increase of Aβ peptides in the brain conforming extracellular amyloid plaques together with intracellular deposits of hyper-phosphorylated tau that form characteristic neurofibrillary tangles. Both pathology hallmarks accompany progressive neuronal loss that ultimately provokes memory loss and severe cognitive dysfunction [1].

AD is an intractable condition to date. The identification of early (preferably pre-symptomatic) biomarkers and true etiologic factors for this condition are the first steps to establish effective primary prevention programs for AD. Consequently, the search for a relatively inexpensive and harmless biomarker for AD continues. Beyond the neuropsychological assessment which still represents the most essential tool for AD and mild cognitive impairment (MCI) screening in humans[2], the most reputed biomarkers for AD are cerebral-spinal fluid (CSF) Aβ42 and phosphorylated-tau protein levels, hippocampal volume measured by magnetic resonance imaging (MRI) techniques and positron electronic tomography (PET) scan with brain Aβ radiotracers. These techniques represent the most studied methods for the detection of prodromal AD. However, there are also drawbacks for each one. For example, although MRI sensitivity showed high sensitivity at baseline, MRI specificity is not unexpectedly limited for MCI conversion to dementia[3] . Furthermore, MRI is restricted to patients with pacemakers. On the other hand, CSF measurements are sometimes variable and imprecise, since measurements vary between studies and laboratories, standardization of analytical as well as pre-analytical procedures will be essential[4]. Some subjects may be unwilling to undergo a lumbar puncture or may have contraindications, such as use of anticoagulants. Finally, something that must be considered is the expense associated with performing amyloid PET scans in large numbers of subjects. Furthermore, its sensitivity and specificity for MCI conversion would require further evaluation, due to a need for improved clinical diagnosis of those subjects with major risk of conversion to dementia, and standardized protocols of data acquisition and imaging analyses. Therefore, opportunities for diagnosis improvement in prodromal or even pre-symptomatic AD still remain. 

In spite of an intensive worldwide research, there is not a definitive plasma or blood biomarker indicating high/low risk of conversion to Alzheimer’s disease to date[5]. Because of their involvement in the generation of amyloid deposits in the brain, the Aβ levels in blood have been widely investigated as potential markers for AD. However, regarding plasma measurements, contradicting results have been reached by using different molecular detection methods or research designs[6]. Beyond the role of plasma Aβ levels as potential AD biomarkers, there are a number of publications suggesting an association between plasma Aβ levels and blood pressure[7-9], body mass index (BMI)[10-12], and different biochemical blood parameters such as insulin levels[13], creatinine [14-18], cystatin C[18,19] or homocystein levels [15,16]. These last observations are of importance because they might help understand the true physiological meaning of APP derived peptides in different tissues. 

Our group is actively involved in the development of novel ELISA sandwich colorimetric tests for detection of Aβ using whole blood instead of plasma alone [20,21]. In fact, using novel technology, we conducted a trial, called the AB128 project, which studied the efficacy of Aβ blood levels as an AD biomarker. Specifically, we found statistical significant differences of some measurements in different blood compartments when comparing healthy controls (HC) and MCI subjects [20]. In spite of these achievements, a definitive conclusion will require independent and extensive replication. Following this idea, a novel trial doubling sample size, the AB255 project, and independent replication efforts using samples obtained from reputed cross-sectional studies are underway to corroborate previous findings.

The aim of the present work is to identify the medical relevant variables correlated with blood Aβ measurements obtained using these novel ELISA techniques. So, we have conducted an unsupervised search of such correlates in the available dataset obtained from the AB128 project. Our study confirmed several variables previously associated with plasma levels of Aβ 40 and pointed to others that seem specific for the amyloid retained in the cell pellet. The information provided herewith can help to define the critical set of covariates for future studies and might provide novel insights in the peripheral function of Aβ.

## Results

First, we analyzed common demographics and clinical differences between the three phenotypic groups (HC, MCI and AD) included in this study (for details see Methods section and table 1). Demographic analysis revealed important differences in age, education, plasma homocysteine levels and *APOE* allele ԑ4 carrier status (dominant model) (Bonferroni corrected p<0.002). However, no significant differences on gender, body mass index, creatinine plasma levels, heart rate, systolic blood pressure, diastolic blood pressure antihypertensive or statin treatment usage was observed. Strong differences on DA and RP Aβ levels were also detected among groups (p<0.001) as observed in a previous report[20].

**Table 1:** Demographics and clinical characteristics of subjects studied.

**Variable**	**Healthy Control**	**MCI**	**AD**
**No. Of subjects**	53	36	51
Age*, years	60.3 (8.3)	75.1 (6.4)	78.3 (5.7)
Education*(%, >8 years)	92.5	30.6	35.3
**Gender (% males)**	34	25	31.4
APOE* (% ɛ4 allele)	18.9	52.8	64.7
**Creatinine (mg/dl)**	0.76(.11)	0.83(.19)	0.86(.21)
Homocysteine* (µmol/L)	9(3.7)	13(4.2)	13.5(3.9)
**Body Mass Index (BMI, kg/m^2^)**	26.6(4)	26.9(3.7)	26.7(4)
**Heart Rate (l/min)**	70.1(10)	70.4(11)	69.4(9)
**Systolic blood pressure (mmHg)**	134.5(21)	144.6(18)	144.5(21)
**Diastolic blood pressure (mmHg)**	79.6(10)	77.8(9)	76.5(10)
**Hypertension treatment (%)**	18.9	27.8	54.9
**Statins treatment (%)**	10.8	19.4	27.5
DA aβ-40* (pg/ml)	44.4(14)	58.9(16)	51(16)
**DA aβ-42 (pg/ml)**	13(12)	14(18)	10.8(7.5)
RP aβ-40* (pg/ml)	84.5(16)	95.5(18.9)	95.5(18.1)
**RP aβ-42 (pg/ml)**	46.5(28)	54.0(46)	51.5(25)
**CP aβ-40 (pg/ml)**	59.4(9.6)	55.2(13.5)	60.8(11.3)
**CP aβ-42 (pg/ml)**	149.1(69)	151.9(70)	165.2(67.9)
**Total amyloid in blood (pg/ml)**	339.7(90)	356.7(101)	373(78.2)

For continuous variables the reported quantities are mean values with standard deviations in brackets.

* P-value<0.002 (Bonferroni Corrected statistical significant threshold)

 Having these findings, we decided to start the covariate search for blood Aβ by applying unsupervised searches in all phenotypic groups together (table S1a,b). Our study indicated that age, creatinine plasma levels and homocysteine were significantly correlated with DA and RP Aβ 40 levels but not with CP Aβ 40 or Aβ 42 in any blood compartment (table 2). The study also suggested a significant, although not high, correlation between hematocrit, uric acid and diastolic blood pressure to different blood Aβ measurements in blood. Interestingly, only serum levels of immunoglobulin A can be nominally correlated to blood beta- Aβ measures in cell pellet (CP) (table 2).

**Table 2:** Most significant correlations between beta-amyloid measurements and clinical parameters observed in this study.

		**DA Aβ40**	**DA Aβ42**	**RP Aβ40**	**RP Aβ42**	**CP Aβ40**	**CP Aβ42**	**Total amyloid**
**AGE**	**Pearson's r**	.370**	.058	.341**	.111	.114	.035	.151
**(n=140)**	**p-value (2-tailed)**	6.73E-06	.495	3.74E-05	.192	.181	.682	.074
	**CI (95%)**	[0.208 - 0.512]	[-0.119 - 0.231]	[0.176 - 0.487]	[-0.066 - 0.281]	[-0.063 - 0.284]	[-0.210 - 0.142]	[-0.026 - 0.318]
**Creatinine (mg/dl)**	**Pearson's r**	.599**	.153	.560**	.112	.087	.025	.188*
**(n=124)**	**p-value (2-tailed)**	2.06E-13	.090	1.42E-11	.216	.339	.780	.036
	**CI (95%)**	[-0.473 - 0.701]	[-0.023 - 0.320]	[0.426 - 0.67]	[-0.050 - 0.292]	[-0.090 - 0.259]	[-0.151 - 0.200]	[0.013 - 0.352]
**DBP (mmHg)**	**Pearson's r**	-.093	-.102	-.127	-.181*	.013	-.130	-.190*
**(n=140)**	**p-value (2-tailed)**	.275	.232	.133	.032	.880	.127	.024
	**CI (95%)**	[-0.264 - 0.084]	[-0.273 - 0.075]	[-0.296 - 0.050]	[-0.346 - 0.005]	[-0.136 - 0.188]	[-0.299 - 0.047]	[-0.354 - -0.015]
**Hematocrit (%)**	**Pearson's r**	-.305**	-.022	-.288**	-.104	.044	.124	.002
**(n=124)**	**p-value (2-tailed)**	.001	.807	.001	.252	.630	.171	.981
	**CI (95%)**	[-0.456 - -0.136]	[-0.197 - 0.154]	[-0.441 - 0.118]	[-0.275 - 0.073]	[-0.133 - 0.218]	[-0.053 - 0.293]	[-0.178 - 0.228]
**Homocysteine (mcmol/L)**	**Pearson's r**	.325**	.069	.345**	.097	.019	.070	.164
**(n=124)**	**p-value (2-tailed)**	2.28E-04	.444	8.63E-05	.286	.837	.439	.069
	**CI (95%)**	[0.158 - 0.474]	[-0.242 - 0.108]	[0.18 - 0.491]	[-0.080 - 0.268]	[-0.157 - 0.194]	[-0.107 - 0.243]	[-0.012 - 0.330]
**Serum Immunoglobulin A (mg/dL)**	**Pearson's r**	.130	.149	.063	.125	.180*	.221*	.253**
**(n=123)**	**p-value (2-tailed)**	.149	.099	.485	.166	.045	.014	.005
	**CI (95%)**	[-0.047 - 0.299]	[-0.028 - 0.316]	[-0.114 - 0.236]	[-0.052 - 0.294]	[0.004 - 0.345]	[0.047 - 0.382]	[0.081 - 0.41]
**Urea (mg/dl)**	**Pearson's r**	.398**	.069	.319**	.074	-.006	-.012	.083
**(n=124)**	**p-value (2-tailed)**	4.74E-06	.447	3.11E-04	.412	.947	.891	.357
	**CI (95%)**	[0.239 - 0.536]	[-0.242 - 0.108]	[0.152 - 0.468]	[-0.103 - 0.247]	[-0.182 - 0.170]	[-0.187 - 0.164]	[-0.094 - 0.255]
**Uric acid (mg/dl)**	**Pearson's r**	.285**	.149	.259**	.049	.165	.000	.093
**(n=124)**	**p-value (2-tailed)**	.001	.099	.004	.591	.067	1.000	.304
	**CI (95%)**	[0.115 - 0.439]	[-0.028 - 0.316]	[0.087 - 0.416]	[-0.128 - 0.223]	[-0.011 - 0.331]	[-0.176 - 0.176]	[-0.084 - 0.264]

*p<0.05 (2-tailed). **P<.01

Previous studies have suggested a strong correlation between age and Aβ levels, as well as between creatinine levels and Aβ levels[14,17,18]. Other studies also indicated sexual dimorphism in amyloid measurements between men and women[17]. The demographic analysis suggested differences in age, *APOE* genotype and education among phenotypic groups. Moreover, the co-linearity between many parameters during unsupervised analysis was also observed, especially true for creatinine, homocysteine, uric acid and urea plasma levels (data not shown). Considering these observations, we decided to conduct a partial correlation analysis of candidate covariates, using an adjusted model including age, gender, phenotypic group, and creatinine levels (table 3). By applying this relatively simple model, we found that most of the correlates between blood Aβ determinations and the other analyzed parameters disappeared, with the exception of DBP which maintained its correlation with RP Aβ 40 (r=-0.18; p=0.046; table 3). Remarkably, an improved correlation between DBP and DA Aβ 40 levels was observed using partial correlations analysis. This specific observation reached a nominally significant association (r=-0.19; p=0.034; table 3). Also, hematocrit correlations with RP Aβ 40 (r=-0.29; p=0.001) and DA Aβ 40 (r=-0.23; p=0.01) remains.

**Table 3:** Partial correlations between Aβ measurements and clinical parameters observed in this study adjusted by Age, creatinine, gender and phenotype.

		**DA Aβ40**	**DA Aβ42**	**RP Aβ40**	**RP Aβ42**	**CP Aβ40**	**CP Aβ42**	**Total amyloid**
**DBP**	**Pearson's r**	-.195	-.130	-.183	-.171	-.045	-.061	-.148
**(n=124)**	**p-value (2-tailed)**	.032	.156	.046	.062	.627	.511	.108
	**CI (95%)**	[-0.358 - -0.020]	[-0.299 - -0.047]	[-0.348 - -0.007]	[-0.324 - 0.019]	[-0.219 - 0.132]	[-0.234 - 0.116]	[-0.316 - 0.029]
**Hematocrit (%**)	**Correlation**	-.288	-.044	-.231	-.130	.071	.112	.010
**(n=124)**	**p-value (2-tailed)**	.001	.631	.011	.157	.442	.225	.910
	**CI (95%)**	[-0.441 - -0.118]	[-0.218 - 0.133]	[-0.437 - -0.057]	[-0.299 - 0.103]	[-0.106 - 0.244]	[-0.065 - 0.282]	[-0.166 - 0.185]
**Homocysteine (mcmol/L**)	**Pearson's r**	-.049	-.008	.041	.012	-.069	.054	.044
**(n=124)**	**p-value (2-tailed)**	.598	.928	.657	.900	.455	.557	.630
	**CI (95%)**	[-0.223 - 0.128]	[-0.184 - 0.168]	[-0.136 - 0.215]	[-0.164 - 0.187]	[-0.242 - 0.108]	[-0.123 - 0.228]	[0.286 - 0.571]
**Serum Immunoglobulin A (mg/dL**)	**Pearson's r**	.019	.120	-.044	.081	.158	.207	.207
**(n=123)**	**p-value (2-tailed)**	.839	.192	.637	.376	.085	.023	.023
	**CI (95%)**	[-0.194 - 0.157]	[-0.057 - 0.290]	[-0.218 - 0.133]	[-0.096 - 0.253]	[-0.018 - 0.325]	[0.032 - 0.369]	[0.032 - 0.369]
**Urea (mg/dl)**	**Pearson's r**	.146	-.012	.059	.004	-.061	-.032	-.022
**(n=124)**	**p-value (2-tailed)**	.111	.894	.519	.969	.508	.728	.809
	**CI (95%)**	[-0.031 - 0.314]	[-0.187 - 0.164]	[-0.118 - 0.232]	[-0.172 - 0.180]	[-0.234 - 0.116]	[-0.207 - 0.145]	[-0.197 - 0.154]
**Uric Acid (mg/dl**)	**Pearson's r**	-.074	.037	-.056	-.056	.160	-.040	-.040
**(n=124**)	**p-value (2-tailed)**	.424	.690	.546	.546	.081	.663	.661
	**CI (95%)**	[-0.247 - 0.103]	[-0.140 - 0.211]	[-0.230 - 0.121]	[-0.230 - 0.121]	[-0.016 - 0.327]	[-0.214 - 0.137]	[-0.214 - 0.137]

These results pointed out that in addition to age, gender, creatinine, phenotype, the hematocrit and DBP could be interesting covariates for DA or RP blood Aβ 40 levels. In contrast, neither of them seemed important for blood Aβ 42 determinations (DA, RP or CP fractions) nor CP Aβ 40 (table 3).

**Figure 1:**
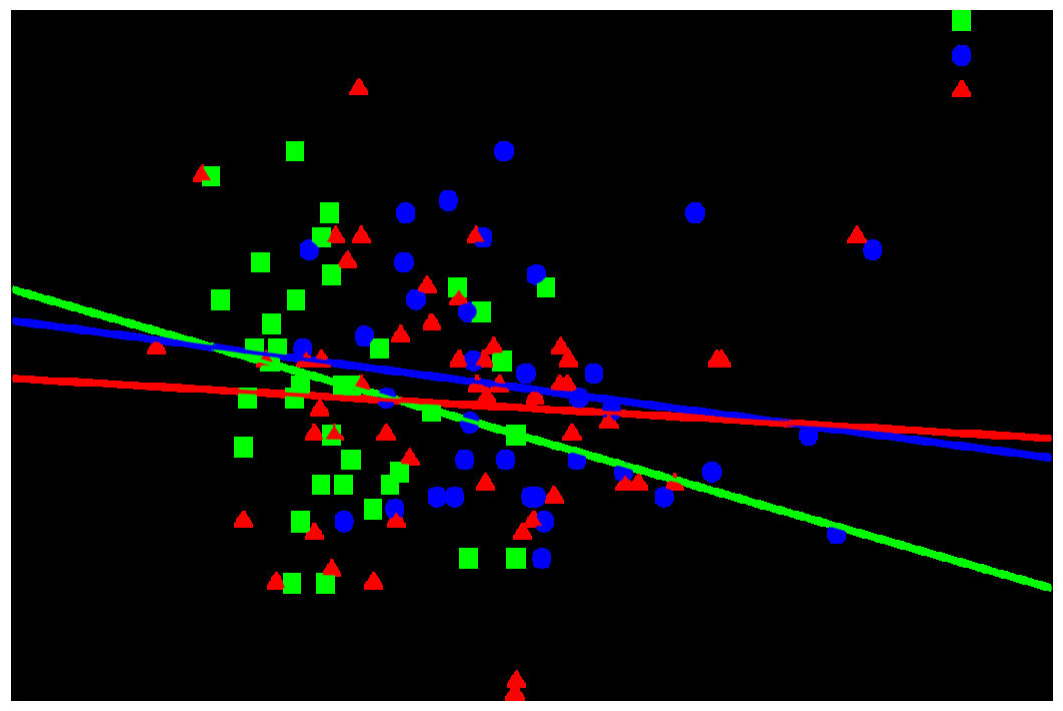
Linear regression between diastolic blood pressure (DBP) volume and levels of DA Aß1-40 in Alzheimer’s disease patients (AD, red triangles), mild cognitive impairment subjects (MCI, blue circles) and healthy individuals (HC, green squares).

Because age, anti-hypertensive drug usage and individual renal function differences among groups may severely distort DBP/DA Aβ 40, or DBP/RP Aβ 40 relationships, we decided to conduct backward linear regression analyses in the three separate phenotypic groups (AD/MCI/HC). The main objective of this supplemental study was to ascertain whether there is a homogeneous correlation between DBP and DA Aβ in plasma (in terms of effect size and direction) in separate groups. Figure 1 shows linear regression between diastolic blood pressure (DBP) volume and levels of DA Aß 40 in the three groups separately. The analysis also permitted to check whether or not DA Aβ 40 can be retained in best regression models after extensive co-variation with well recognized parameters affecting DBP in elderly subjects (i.e. anti-hypertensive drug usage, BMI, age, gender, renal function variables, APOE genotype, etc.). Importantly, our results suggested a consistent correlation effect of DA Aβ 40 levels in three phenotypic groups (table 4). Most importantly, in spite of including an extensive number of covariates, DA Aβ 40 was retained in the best backward regression model of the three phenotypic groups. This last result supports the notion that plasma Aβ 40 levels/DBP correlation was independent of well-established physiological and pathological parameters affecting blood pressure in elderly subjects. Furthermore, the association between both variables was important enough to be selected as a critical one irrespective of cognitive status of studied individuals (table 4).

**Table 4:** Backward linear regression analysis exploring factors correlated with diastolic blood pressure.

			**Unstandardized Coefficients**		**Standardized Coefficients**		**95.0% Confidence Interval for B**	**95.0% Confidence Interval for B**
**Groups**	**Model**		**B**	**Std. Error**	**Beta**	**Sig.**	**Lower Bound**	**Upper Bound**
**Group 1**		(Constant)	60.755	6.090		.000	48.455	73.054
**Model 9**		DA Aβ40	-.261	.092	-.481	.007	-.447	-.075
**AD**		Creatinine	23.416	7.282	.559	.003	8.710	38.121
		APOE	5.124	2.411	.280	.040	.254	9.994
		Statins	4.060	2.494	.211	.111	-.977	9.097
		Vitamin B12	.010	.006	.241	.071	-.001	.021
**Group 2**		(Constant)	38.873	15.731		.019	6.788	70.957
**Model 10**		DA Aβ40	-.318	.139	-.551	.030	-.602	-.034
**MCI**		Creatinine	30.277	13.790	.627	.036	2.152	58.401
		Sex	7.632	4.365	.363	.090	-1.269	16.534
		BMI	.708	.387	.290	.077	-.081	1.497
**Group 3**		(Constant)	4.741	16.357		.774	-28.538	38.021
**Model 11**		DA Aβ40	-.233	.126	-.263	.074	-.489	.024
**Healthy Controls**		Cholesterol	.169	.049	.468	.002	.069	.270
		Hematocrit	1.100	.290	.499	.001	.510	1.690

Backward linear regression analyses also suggested that variables retained in the best model (and significantly affecting DBP) in the three phenotypic groups are different (table 4). In fact, cardiovascular factors such as total cholesterol levels and hematocrit seem much more important for DBP in the healthy (and younger) control group than in the MCI or AD groups. On the contrary, APOE genotype and creatinine levels seemed to only correlate well with the AD status. Remarkably, DA Aβ40 remains as one of the selected variables in the MCI group (table 4).

## Discussion

The discovery of easily accessible, cost-effective and pre-symptomatic biomarkers for AD is mandatory to fuel secondary prevention strategies. There are numerous efforts underway to reach this objective[5]. Technologies under investigation are multiple and not only related to amyloid levels. In fact, promising results are emerging by applying novel technologies such as analyses of differences in gene expression patterns[22,23], micro-RNA analyses [24] or proteomic-based approaches among others[25]. 

The efficacy of Aβ blood levels on AD prediction has also been recursively investigated. Original investigations suggested an association between Aβ levels and AD, although results were not uniform among studies[6,8,9,19-21,26-28]. Thus, preliminary findings have remained controversial and would need independent validations [6,19]. Notably, the direct comparison between most studies is almost impossible due to the existence of many different immunodetection methods, including different polyclonal antibodies, differences on study designs or the lack of standardization or consensus in the analysis model. Therefore, the methods’ differences in previously published studies prevent a direct comparison among currently available results or meta-analysis.

In the present study, the Araclon’s blood Aβ immunodetection system permitted the identification of previously observed well-established correlations, including blood pressure, hematocrit, creatinine and homocysteine levels. These observations are of importance mainly for two reasons: ( i) on the one hand, the identification of similar correlations to those previously reported by independent research groups using our technology directly reassured the validity of our methods; (ii) on the other hand, the identification of such covariates may serve to a better design of future larger studies by controlling critical covariates affecting Aβ levels.

Based on previous investigations and on the findings presented here, variables such as age, gender and renal function biomarkers (i.e. serum creatinine levels) appeared as the most important covariates correlated to the levels of plasma (DA or RP) Aβ 40. The effect of these covariates in the blood levels of Aβ 42 appear to be smaller in our study. However, taking into account previous results, the incorporation of such covariates to future analyses also seems pretty reasonable for studies with Aβ 42. This could seem like an apparently obvious recommendation. In fact, it has been previously suggested by others[14]. However, only a minority of studies such as the cardiovascular health study (CHS) has incorporated renal function biomarkers in their models [19]. 

In contrast, the rest of correlates initially detected seem not to be independent of creatinine levels. Thence, we suggest that urea, uric acid and homocysteine associations have appeared only as a consequence of strong creatinine/ Aβ relationship as previously mentioned. Consequently, it makes no sense to incorporate them to regression models due to high co-linearity among them. 

An exception to this rule might be the hematocrit, which retained its correlation with Aβ 40 levels even after the adjustments of age, gender, phenotype and plasmatic creatinine levels. It is possible that the hematocrit fraction is somehow affecting the equilibrium between cell-bound and cell-free in plasma amyloid. However, this association was not uniform enough among the phenotypic groups (HC, MCI and AD) to delineate a definitive conclusion. A replication increasing sample size is necessary to corroborate hematocrit findings. Interestingly, these results are consistent with previous findings in the Japanese population suggesting a genuine correlation between both physiological parameters[7]. Although some authors have speculated on hematocrit implication on cognition performance[29], its role on AD etiology is not well understood and deserves further investigation. 

On the contrary, blood pressure (BP) is a well-recognized risk factor for dementia. Indeed, AD has been linked to cardiovascular risk factors, such as diabetes mellitus, hyperlipidemia, and, in particular, elevated BP[9]. DBP/Aβ 40 correlations appear consistent in our study irrespective of the individual’s phenotype. This observation corroborated, in terms of effect size and direction, previous reports [8,30]. The largest study reported to date on blood pressure and plasma Aβ levels was held by Lambert et al. in their “Three-Cities Study”[8]. The authors suggested associations between the Aβ 40/ Aβ 42 ratio and the systolic blood pressure, the DBP or the hypertension. However, they also pointed out that the observed effect could be mainly driven by plasma Aβ 40. Furthermore, the mechanism underlying this preferential association could be related to the Aβ 40 peptide’s properties on vascular vessels (for details, see Lambert et al. and references therein). Concretely, previous studies have indicated that Aβ 40 peptides could modify cerebral blood vessels in vitro and induce a decrease of cerebral flow and cerebral blood volume in vivo[31,32] and that Aβ 40 has important effects on vasoconstriction[32]. Our results independently confirm the importance of Aβ 40 in blood pressure in humans. Most importantly, the stratified analysis suggests that this effect can be detected in elderly patients affected or unaffected by MCI and AD.

The relationships between blood Aβ levels, blood pressure and Alzheimer’s disease have also been explored in the Honolulu Heart Program/Honolulu Asia Aging Study[9]. The authors of that study suggested that the risk for AD significantly increased with lower levels of plasma Aβ 40; hazard ratio: 2.1 [95% CI: 1.4 to 3.1]; and detected evidence of interaction between DBP and plasma Aβ 40. Importantly, low plasma Aβ 40 or 42 was associated with the presence of cerebral amyloid angiopathy but not with the other neuropathologies. Therefore, the disruption of blood pressure homeostasis in midlife could contribute to future risk of dementia. Therefore, reduced levels of Aβ 40 in midlife could be directly or indirectly involved in the early pathogenic process of AD. 

A major limitation of this study is the observed differences in demographic characteristics among groups. In general, healthy controls are younger and with higher educational scores than MCI or AD subjects. However, despite this limitation the study was able to replicate previous findings as already pointed out, although the new insights shown here would deserve a further replication. 

Another limitation would be the small sample size. This might explain the discordance observed in the relationships between amyloid measurements in different compartments and physiological parameters studied (Table 2 and 3). For example, backward regression analyses using Aβ40 RP fraction versus DBP were not fully consistent with the results obtained in Aβ40 DA compartment (data not shown). This divergence might be attributable to having a small sample size which in turn may provoke random chance oscillations during effect size estimation. Alternatively, unknown factors could be affecting peptide measurements in different compartments. For this reason, it would be advisable to independently replicate these results to corroborate these findings.

Finally, the biological meaning of these findings cannot be ignored because it could provide essential information about the real physiology of APP derived peptides in different human systems. For instance, the creatine/creatinine energy cycle in brain and muscle could have a closer relation with amyloid physiology than previously anticipated [33]. Therefore, observed correlations would need a more deep interpretation beyond a mere correlation with the renal function as proposed in previous studies. In contrast, the covariates related to Aβ CP fraction remained almost unknown. In fact, only serum immunoglobulin A levels displayed a weak correlation with the amyloid measured in the cell pellet fraction of the blood. Importantly, the presence of immunoglobulin fragments in the amyloid plaques of the AD brain had been observed[34] and this observation could be easily linked with AD neuroinflammatory hypothesis[35] . However, we considered that this last observation would require independent confirmation in future studies. Nevertheless, the novelty and potential of CP amyloid warrant further investigation.

## Materials and Methods

### Patients

We selected one hundred and forty subjects from the AB128 project. All these patients were recruited at a single clinical research site, the Memory Clinic of Fundació ACE, Institut Català de Neurociències Aplicades, Barcelona, Spain. The original design included three phenotypic groups of individuals: AD patients (n=51; 31.4% males), mild cognitive impairment (MCI) patients (n=36; 25% males) and healthy controls (HC) (n=53; 34% males). The demographic characteristics of the subjects under study are summarized in Table 1. 

AD, HC and MCI criteria used to recruit subjects in this study have been described in our previous works[20,21]. Cognitive assessment was performed according to the routines of the Memory Clinic of Fundació ACE as described elsewhere[36]. Briefly, MCI subjects fulfilled the Petersen’s diagnostic criteria[37] including subjective memory complaint, normal general cognition, preserved performance in activities of daily living, absence of dementia and a measurable impairment in memory function, with or without deficit in other cognitive domains[38]. All MCI subjects had a CDR rating of 0.5. Based on the Wechsler Memory Scale-III (WMS-III) of NBACE battery an impaired delayed verbal recall for which recognition testing did not improve performance, classified MCI amnesic subjects as having an “Encoding/Storage” pattern of memory loss. Diagnosis of AD individuals followed NINCDS-ADRDA criteria[39,40], a CDR of 1 point or more and a Mini-Mental State Examination (MMSE) below 24 points. Healthy controls were cognitively normal when evaluated in the Fundació ACE, had MMSE scores of at least 26 (considering that the MMSE cut-off has been demonstrated to be <25 in the Spanish population [41]) , and also had a normal neuroimaging MRI profile. 

A written informed consent was obtained from every participant. The study’s protocols were reviewed and approved by the Ethical Committee of the Hospital Clinic i Provincial (Barcelona, Spain). 

### Blood sampling, biochemical determinations and Aβ measurements

Blood samples from each participant were routinely processed in Fundació ACE as previously described[21]. Plasma biochemical and hematologic measurements were obtained in a reference laboratory according to routine clinical standards. For blood amyloid testing, all samples were analyzed in triplicate in the same laboratory and blinded for analysts. For each of the three blood fractions analyzed, two specific ELISA sandwich kits, ABtest 40 and ABtest 42 (Araclon Biotech Ltd. Zaragoza, Spain) were used as described elsewhere. Briefly, before analysis, plasma and blood cell samples were pretreated by dilution in a formulated saline buffer with 1% blocking polymer according to the supplier’s instructions. We determined three parameters for both the Aβ40 and Aβ42 peptides in each blood sample. One determination was performed using the undiluted plasma sample, another using the plasma sample diluted 1:3 with the aforementioned formulated buffer, and a third using the cellular pellet that remained after plasma collection. The peptide amount in the undiluted plasma sample corresponds to the directly accessible (DA) peptide. The 1:3 dilution of the plasma was chosen because it provided the maximum peptide recovery from the plasmatic sample. Thus, this determination included the DA peptide and the peptide that was recovered from the plasma matrix (RP). Additionally, the peptide associated with the cellular pellet (CP) was measured in a 1:5 dilution of the pellet that remained after plasma collection.

### Statistical Analysis

#### a) Unsupervised Pearson’s correlation analyses

Aβ 40 and Aβ 42 peptides measurements in three different blood compartments (DA, RP and CP), several calculated indices derived from these primary measurements and thirty seven medical relevant variables obtained from 140 individuals were sorted and arranged in a text file. Variable name, data source, units and main statistical characteristics of each variable are detailed in table 1 and table S1a and S1b. 

To calculate standardized Pearson’s coefficients, the constructed text file was processed using the R statistical package according to programmers’ instructions[42]. R command c*or* was selected for this purpose because it permits automatic (and appropriated) management of missing cells. Pearson’s coefficients of determination (*r* ^*2*^) were easily derived using R calculation tools (table S1b). 

Top correlated variables for each primary measurement plus total Aβ in blood were filtered and ranked using conventional excel spreadsheets (table S1a,b). Selected candidate covariates for each primary measurement were chosen for further research.

#### b) Partial correlation and regression-based analyses

On the basis of Pearson’s coefficients analysis, eight variables (i.e. creatinine (mg/dl), urea (mg/dl), age at baseline (years), homocysteine (mcmol/L), uric acid (mg/dl), serum immunoglobulin A (mg/dL), diastolic blood pressure (DBP) (mmHg) and Hematocrit (%) were selected for further research. We used SPSS 18 package (PASW Statistics for Windows, Version 18.0. Chicago: SPSS Inc.) to re-calculate Pearson’s coefficient of determination (*r* ^*2*^) and statistical significance of all selected variables and blood Aβ measurements. Partial correlation analyses were conducted to calculate adjusted correlation coefficients of each selected variable and Aβ levels. Adjusted covariables for partial correlation analyses used were age, gender, phenotypic group (AD/MCI/HC) and plasmatic creatinine. 

To further demonstrate the independence of DBP/ Aβ relationships with the rest of co-variables or phenotypic status, we conducted a backward regression analysis on each phenotypic group separately. In this specific analysis the choice of predictive variables was carried out by an automatic procedure using SPSS 18 on each phenotypic group. Backward elimination of variables, which involves starting with multiple variables affecting DBP (DA Aβ 40, creatinine, APOE genotype, gender, age, body mass index, antihypertensive treatment, statins treatment, cholesterol levels, triglycerides level, vitamin b12 levels, hematocrit and homocysteine), testing the deletion of each variable using a chosen model comparison criterion, deleting the variable (if any) that improves the model the most by being deleted, and repeating this process until no further improvement is possible. Regression-based analyses of PAD/Aβ were graphically represented using gnuplot 4.6 (URL http://gnuplot.info). 

## Supporting Information

Table S1 **A: Pearson coefficients (**r**) between plasma amyloid levels and medical variables.** B: Pearson coefficients (r^2^) between plasma amyloid levels and medical variables.
(XLSX)
