# Correction: Blood Amyloid Beta Levels in Healthy, Mild Cognitive Impairment and Alzheimer’s Disease Individuals: Replication of Diastolic Blood Pressure Correlations and Analysis of Critical Covariates

**DOI:** 10.1371/annotation/0f80896d-487c-4323-8eeb-774b6d517005

**Published:** 2014-01-02

**Authors:** Agustín Ruiz, Pedro Pesini, Ana Espinosa, Virginia Pérez-Grijalba, Sergi Valero, Oscar Sotolongo-Grau, Montserrat Alegret, Inmaculada Monleón, Asunción Lafuente, Mar Buendía, Marta Ibarria, Susana Ruiz, Isabel Hernández, Itziar San José, Lluís Tárraga, Mercè Boada, Manuel Sarasa

Figure 1 is incorrect. Please see the correct Figure 1 here: http://plosone.org/corrections/pone.0081334.g001.cn.tif 

